# Placental 5-methylcytosine and 5-hydroxymethylcytosine patterns associate with size at birth

**DOI:** 10.1080/15592294.2015.1062963

**Published:** 2015-06-19

**Authors:** Chinthika Piyasena, Rebecca M Reynolds, Batbayar Khulan, Jonathan R Seckl, Gopi Menon, Amanda J Drake

**Affiliations:** 1 University/British Heart Foundation Center for Cardiovascular Science; University of Edinburgh; The Queen's Medical Research Institute; Edinburgh, UK; 2 Neonatal Unit; Simpson Center for Reproductive Health; Royal Infirmary of Edinburgh; Edinburgh, UK

**Keywords:** 5-methylcytosine, 5-hydroxymethylcytosine, birth weight, imprinted gene, Placenta

## Abstract

Altered placental function as a consequence of aberrant imprinted gene expression may be one mechanism mediating the association between low birth weight and increased cardiometabolic disease risk. Imprinted gene expression is regulated by epigenetic mechanisms, particularly DNA methylation (5mC) at differentially methylated regions (DMRs). While 5-hydroxymethylcytosine (5hmC) is also present at DMRs, many techniques do not distinguish between 5mC and 5hmC. Using human placental samples, we show that the expression of the imprinted gene *CDKN1C* associates with birth weight. Using specific techniques to map 5mC and 5hmC at DMRs controlling the expression of CDKN1C and the imprinted gene *IGF2*, we show that 5mC enrichment at KvDMR and DMR0, and 5hmC enrichment within the *H19* gene body, associate positively with birth weight. Importantly, the presence of 5hmC at imprinted DMRs may complicate the interpretation of DNA methylation studies in placenta; future studies should consider using techniques that distinguish between, and permit quantification of, both modifications.

## Introduction

An abundance of evidence from human and animal studies has shown that low weight at birth is associated with an increased risk of cardiometabolic and neuroendocrine disease in later life.^1^ The factors that determine size at birth are complex but include differences in placental size and function.^2^ Imprinted genes, which are preferentially expressed from one parental allele only, are of particular importance in determining normal placental growth and transfer capacity, notably transport of the nutrients essential for normal fetal growth.^3^ Evidence from mouse models and humans suggests that genes that are paternally expressed tend to increase fetal growth, whereas maternally expressed genes restrict fetal growth. The expression of imprinted genes is largely regulated by epigenetic mechanisms, particularly DNA methylation (5-methylcytosine; 5mC) at differentially methylated regions (DMRs). Aberrant imprinting leads to abnormalities of both placental and fetal growth in animal models.^4-7^ In addition, altered placental expression of imprinted genes has been reported in association with human fetal growth restriction.^8-10^

The imprinted *insulin-like growth factor 2* (*IGF2*) gene has a major role in the matching of placental nutrient supply to fetal demand,^4^ and altered IGF2 expression has been reported in association with fetal growth restriction in humans.^9,10^ *IGF2*, and the neighboring *H19* gene, are situated in an imprinted gene cluster on human chromosome 11p15. Expression of the paternally expressed IGF2 and the maternally expressed H19 is controlled by differential methylation at imprinting control region 1 (ICR1),^11,12^ which contains binding sites for the zinc-finger CCCTC-binding factor (CTCF) protein. DNA methylation at ICR1 on the paternal allele prevents CTCF binding and is permissive for *IGF2* gene expression. In contrast, CTCF can bind the unmethylated maternal ICR1 and act as an insulator, blocking the access of *IGF2* promoters to the *H19* downstream enhancers, resulting in the activation of *H19* expression. Loss of imprinting at the IGF2/ICR1/H19 domain has been reported in some cases of Beckwith-Wiedemann syndrome (BWS), which is associated with prenatal overgrowth, and with Silver-Russell syndrome (SRS), which is associated with profound pre- and post-natal growth failure.^13^ Approximately 50% of growth-restricted individuals with SRS cases show loss of methylation at ICR1, which could lead to decreased IGF2 expression and consequent growth restriction.^8,14^ IGF2 expression is additionally modulated by DNA methylation at a number of other DMRs, ^15^ and altered placental and umbilical cord 5mC at DMRs controlling IGF2 expression has been reported in association with fetal growth restriction in some,^16-19^ but not all^15,20^ studies.

The maternally expressed *cyclin dependent kinase 1C* (*CDKN1C* or *p57*^*KIP**2*^) gene, located on the centromeric domain of the chromosome 11p15 imprinting cluster, encodes a cyclin dependent kinase inhibitor that induces cell cycle arrest and is a major regulator of fetal growth.^21,22^ CDKN1C is expressed at high levels in the placenta and its expression is regulated by DNA methylation at a CpG island (KvDMR), which is normally methylated on the maternal allele.^23^ In mice, *cdkn1c* knockout or loss of function mutations resulting in enhanced cell differentiation and proliferation have been used to model BWS.^22,24^ While mutations at *CDKN1C* occur in a small proportion of individuals with BWS,^25^ in the majority of cases, there is loss of methylation at KvDMR^14,26,27^ with associated silencing of *CDKN1C*. The converse findings have been reported in SRS.^28^

5-hydroxymethylcytosine (5hmC) is a recently described cytosine modification that may be an intermediate in the DNA demethylation pathway ^29^ and is present in the placenta.^30^ Since commonly used methods of assessing DNA methylation using bisulfite conversion do not discriminate between 5mC and 5hmC, ^31^ previous studies reporting altered DNA methylation at placental DMRs in association with fetal growth may be confounded. We used specific enrichment techniques to map 5mC and 5hmC at the DMRs controlling the expression of IGF2 and CDKN1C in human placenta, and additionally explored relationships between 5mC, 5hmC, gene expression, and fetal growth across the full range of normal birth weight.

## Methods

### Cohort

Placental samples were collected from 72 women with a singleton pregnancy at term (>37 weeks) under the Edinburgh Reproductive Tissue Bio-bank (ERTBB). Participants provided full informed, written consent. The ERTBB works with ethical and governance approval from the Scottish Academic Health Sciences Collaboration (SAHSC), Human Annotated BioResource (East of Scotland Research Ethics Service 13/ES/0126) and, previously, the West of Scotland Research Ethics Committee 4 (09/S0704/3). Samples were collected at delivery by trained staff from the center of the placenta (fetal side), away from the umbilical cord and large vessels. Samples were snap-frozen or placed in RNAlater with storage at −80°C. Pregnancies complicated by congenital abnormalities or diabetes were excluded. The sample comprised babies with the full range of birth weight [mean 3467 g (SD 829 g)], including small for gestational age [SGA, n = 21, birth weight <10^th^ percentile, birth weight SD score mean −1.85 (SD 0.44)], appropriate for gestational age [AGA, n = 24, birth weight 10^th^−90^th^ percentiles, birth weight SD score mean −0.36 (SD 0.79)], and large for gestational age (LGA, n = 27, birth weight >90^th^ percentile, birth weight SD score mean 1.97 (SD 0.35)].

### Gene expression

Total RNA was extracted from placenta using the Qiagen RNeasy Fibrous Tissue Minikit (QIAGEN, Crawley, UK) following the manufacturer's instructions. High quality RNA was obtained from 64 samples and the characteristics of these 64 did not differ from the whole dataset. RNA (1 µg) was reverse transcribed using the Promega Reverse Transcription system (Promega, Southampton, UK) and the expression of the imprinted genes *IGF2*, *H19* and *CDKN1C* was quantified using real-time PCR using pre-designed assays (*IGF2* and *CDKN1C*) (Applied Biosystems, Warrington, UK), the Roche UPL System (YWHAZ) or SYBR green technology (H19) (Roche, Burgess Hill, UK) using a Roche LightCycler® 480. YWHAZ was chosen from a panel as the most stable reference gene using Normfinder^32^ and geNorm^PLUS^ as part of the qbase^PLUS^ software (Eclipse v 3.7, Biogazelle, Zwijnaarde, Belgium). See **Table 1** for primer sequences.

**Table 1.** Primer sequences for real-time PCR. Primers for *GAPDH* and the *H19* gene body taken from.^30^ Coordinates refer to UCSC genome browser hg18

Gene expression	
Gene	Assay details
Cyclin-dependent kinase inhibitor 1C (CDKN1C)	Applied Biosystems: Hs00178938_m1
Insulin like growth factor 2 (IGF2)	Applied Biosystems: Hs00171254_m1
H19	F:AACCCACAACATGAAAGAAATGG
	R: AGAGGGTTTTGTGTCCGGATT
Tyrosine 3-monooxygenase/tryptophan 5-mono-oxygenase activation protein, zeta polypeptide (YWHAZ)	F:GATCCCCAATGCTTCACAAG
	R:TGCTTGTTGTGACTGATCGAC
	UPL Probe no. 30
**5mC/5hmC enrichment**	**Assay details**
GAPDH 30	F:CGGCTACTAGCGGTTTTACG
chr12:6513796-6513984	R:AAGAAGATGCGGCTGACTGT
H19 gene body 3 30	F: CTGGTGCTCACCTTCCAGAG
chr11:1973671-1973802	R: ATGGTGCTACCCAGCTCAAG
H19 gene body 4 30	F: GCCAGCTACACCTCCGTTG
chr11:1975242-1975378	R: AGCTAGGGCTGGAAAGAAGG
H19 gene body 1 30	F: CTCAGCTCTGGGATGATGTG
chr11:1973443+1973573	R: AGCCCAACATCAAAGACACC
H19 ICR (CTCF 6)	F: AGTTGTGGAATCGGAAGTGG
chr11:1977617-1977807	R: GATAATGCCCGACCTGAAGA
IGF2 DMR0	F: TTTCATATTCCGTGCCATGA
chr11:2125774-2125987	R: CCTGCCTAGAGCTCCCTCTT
IGF2 DMR2	F: CGTTGAGGAGTGCTGTTTCC
chr11:2111194-2111431	R: CACAGCAAGCAAGGAAGTCA
KvDMR	F: TGCGGATTCCAGACTCCAAT
chr11:2676925+2677065	R: GCTCCCATCTGCACCTTATG

### Analysis of 5mC/5hmC

DNA was extracted from all 72 samples using DNeasy Blood & Tissue Kit (Qiagen, Crawley, UK) with RNAse treatment (Sigma-Aldrich, Dorset, UK). DNA was sonicated (200–300 bp fragments for 5mC; 500-600 bp for 5hmC enrichment) using the Covaris E220 Ultrasonicator (Covaris, MA, USA). 5mC enrichment was performed using the Active Motif MethylCollector Ultra kit (Active Motif, La Hulpe, Belgium) as per the manufacturer's instructions. 5hmC enrichment was performed using hydroxymethyl-DNA immunoprecipitation, as previously described.^33^ Immunoprecipitated (ip) and input DNA were purified using the QIAquick PCR Purification Kit (Qiagen, Crawley, UK). Percentage enrichment of 5mC and 5hmC (ip/input) was analyzed at the region of ICR1 containing the sixth CTCF binding site which is most consistently associated with the transcriptional status of both *IGF2* and *H19*;^34^ at 2 other DMRs known to influence expression of IGF2 expression (DMR0, DMR2); and at KvDMR, using real-time PCR with SYBR Green Technology. 5hmC enrichment within the *H19* gene body was also analyzed since previous studies have identified the region as enriched for 5hmC. ^30^ As the ICR1 is known to be enriched for 5mC, the *H19* gene body is enriched for 5hmC and the *GAPDH* promoter is depleted for both modifications;^30^ enrichment at these regions acted as positive and negative controls for each cytosine modification. Primer details are given in **Table 1**.

### Statistics

Weight measurements were adjusted for sex by converting to SD scores (z-scores) and centiles using LMSgrowth version 2.71 (Pan H, Cole TJ. LMSgrowth, a Microsoft Excel add-in to access growth references based on the LMS method. Version 2.71. http://www.healthforallchildren.co.uk/; 2011). The data source was “British 1990 reference data, reanalyzed 2009.” Values for relative gene expression and percentage enrichment were natural log transformed to achieve a normal distribution. Multivariate linear regression analysis was used to test the hypothesis that birth weight is associated with altered mRNA levels and 5mC or 5hmC enrichment. Covariates that could confound the association or be in the causal pathway were added into the model and included maternal body mass index (BMI), parity, smoking, and pre-eclampsia. The outcome variable was birth weight standard deviation (SD) score. Statistical significance was set at *P* < 0.05 (2-sided). Data were analyzed using SPSS version 19 (IBM, New York, USA).

## Results

### Cohort

Characteristics of the mothers and babies are shown in **Table 2**. For illustration, the data is presented as grouped by SGA, AGA and LGA and includes variables used in the regression models. There were differences between groups in birth weight, birth weight centiles and maternal BMI.

**Table 2.** Characteristics of mothers and babies

	SGA (n=21)	AGA (n=24)	LGA (n=27)	*P* values
Birth weight, g, mean (SD)	2515 (201)	3250 (383)	4401 (189)	<0.001
Birth weight centile, mean (SD)	4.38 (3.1)	38.3 (26.4)	96.9 (2.4)	<0.001
Maternal BMI, kg/m^2^, mean (SD)	28.7 (8.2)	23.2 (2.9)	29.9 (6.1)	<0.001
Pre-eclampsia, n (%)	4 (19.1)	0	0	
Hypertension during pregnancy, n (%)	1 (4.8)	0	1 (3.7)	
Smoking during pregnancy, n (%)	4 (19.1)	1 (4.2)	0	
Primiparity, n (%)	7 (33.3)	8 (33.3)	3 (11.1)	

### Gene expression

**Figure 1.**
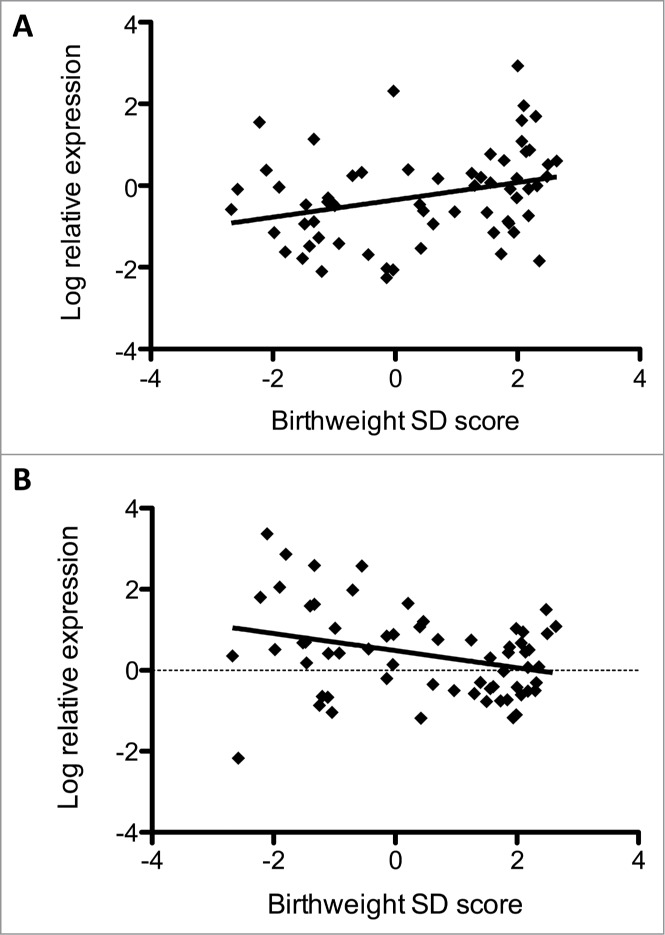
Relationships between gene expression and birth weight. (**A**) Relative expression of IGF2 vs. birth weight SD score and (**B**) relative expression of CDKN1C vs. birth weight SD score.

There was a positive correlation between birth weight SD score and placental IGF2 mRNA levels (*r* = 0.30, *P = *0.017), which remained significant when adjusted for maternal BMI, parity and pre-eclampsia (standardised β = 0.27, *P=*0.038) (**Fig. 1A**). However, the association was lost when maternal smoking was added into the model (standardized β = 0.20, *P = *0.104) or when offspring of mothers who smoked were removed from the model (standardized β = 0.23, *P = *0.085). There was a negative correlation between birth weight SD score and placental CDKN1C mRNA expression (*r* = −0.31, *P = *0.013) (**Fig. 1B**) which remained significant after adjustment for maternal BMI, parity, pre-eclampsia and smoking (standardized β = −0.26, *P* = 0.037) or when women who smoked were removed from the analysis (standardized β = −0.27, *P = *0.043).

### Analysis of 5mC and 5hmC

**Figure 2.**
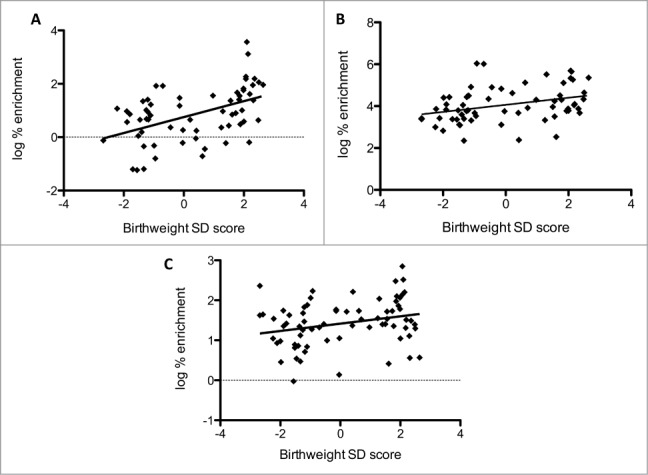
Relationships between 5mC, 5hmC, and birth weight. (**A**) Percentage enrichment of 5mC at *IGF2* DMR0 versus birth weight SD score, (**B**) percentage enrichment of 5mC at KvDMR vs. birth weight SD score and (**C**) percentage enrichment of 5hmC at *H19* gene body vs. birth weight SD score.

5mC enrichment was seen at DMR2, ICR1, and KvDMR with lower levels of enrichment at DMR0 (**Table 3**). 5mC enrichment at DMR0 and KvDMR was independently associated with birth weight SD score in adjusted analyses (**Figs. 2A and B**) (DMR0: *r = *0.37, *P = *0.002 standardized β = 0.27, *P = *0.023; KvDMR: *r = *0.29, *P = *0.021 standardized β = 0.29, *P = *0.016). 5hmC enrichment was identified at all DMRs, notably DMR2 (**Table 3**). 5hmC enrichment within the H19 gene body correlated with birth weight SD score in adjusted analyses (**Fig. 2C**) (*r = *0.27, *P = *0.023; standardized β = 0.22, *P = *0.041). There were no significant relationships between gene mRNA levels and 5mC or 5hmC at any of the regions studied.

**Table 3.** Percentage enrichment of 5mC and 5hmC at individual DMRs.

Locus	5mC % enrichment (ip/input). Mean ± SE	5hmC % enrichment (ip/input). Mean ± SE
GAPDH	0	0.12 ± 0.02
DMR0	3.79 ± 0.64	3.27 ± 0.37
DMR2	28.73 ± 2.15	21.38 ± 2.07
ICR1	42.91 ± 2.24	3.12 ± 0.39
KvDMR	57.16 ± 4.85	1.53 ± 0.13
H19 gene body	Not tested	4.87 ± 0.34

## Discussion

Our results are in agreement with those from a number of previous studies showing the association of poor fetal growth with decreased placental IGF2 expression.^9^ Here, we extend these studies to show that this relationship holds across the range of normal birth weight. Previous studies have reported decreased IGF2 expression in placenta from SGA pregnancies,^9,16^ which may occur from early on in pregnancy,^35^ and studies in mice confirm that IGF2 controls a substantial part of fetal growth.^36^ These data are consistent with the predicted effects of IGF2, since paternally expressed genes augment fetal and placental development. Although the number of women who smoked was small, our data suggest that maternal smoking may explain part of this relationship, since controlling for maternal smoking, or removing smokers from the analysis weakened the association. An effect of maternal smoking on IGF2 expression in placenta would not be surprising since we have recently shown that maternal smoking reduces IGF2 expression in human fetal liver.^37^ Maternally expressed genes including CDKN1C act to restrict fetal and placental growth,^38^ and our data showing decreasing CDKN1C expression with increased birth weight are consistent with this and with a previous study reporting increased expression in association with SGA.^10^ Maternal smoking had no effect on CDKN1C expression, suggesting that any effects of smoke exposure are gene-specific, again consistent with our previous data in human fetal liver.^37^

5mC enrichment at IGF2 DMR0 and KvDMR was positively related to birth weight SD score. However the consequences of this are unclear, since there was no correlation between DNA methylation at these regions and IGF2 and CDKN1C mRNA levels. The lack of correlation between mRNA levels and DNA methylation is consistent with previous studies in umbilical cord and placenta which report altered expression of a number of imprinted genes in the absence of the corresponding expected changes in DNA methylation^20,39^ and suggests that alternative mechanisms may lead to dysregulation of the expression of these genes in the placenta. 5hmC enrichment within the *H19* gene body was also positively related to birth weight SD score. Although 5hmC is about 10-fold less abundant than 5mC across the genome, this varies across tissues and genomic location.^40^ In addition to its role in DNA demethylation, 5hmC is a stable and functional epigenetic mark ^41^ and is enriched in the bodies of actively transcribed genes, at a number of transcription start sites, and at CTCF binding sites.^30,42,43^ We identified significant enrichment of 5hmC at DMR2 and, although 5hmC is also known to be present at some imprinted DMRs, including across *IGF2-H19*, its precise role at these loci is unclear.^30^ However, studies that show that H19 remains highly enriched for 5hmC in cell culture despite the global loss of 5hmC elsewhere,^43^ and that such loci are also protected from demethylation in the post-fertilization embryo,^44^ suggest that 5hmC dynamics at imprinted loci may differ from those in the remainder of the genome. Additionally, since 5hmC may be an intermediate in the DNA demethylation pathway, 5hmC at DMRs could result from the conversion of 5mC during the demethylation process.^29^

Both 5mC and 5hmC are resistant to deamination by bisulfite conversion, so that the cytosine methylation quantified using bisulfite conversion represents the sum of 5mC and 5hmC levels.^31^ Although the techniques used in our study do not permit us to compare the relative enrichment of 5mC and 5hmC quantitatively, the presence of 5hmC within DMRs in imprinted regions in human placenta mean that studies of DNA methylation using conventional bisulfite conversion might overestimate 5mC levels at DMRs, particularly if the results of such studies are used to infer potential effects of altered methylation on gene expression. Our findings of significant enrichment of 5hmC and a lower enrichment of 5mC at DMR2 relative to the other DMRs analyzed suggest that studies using bisulfite conversion techniques that report small changes in cytosine methylation at DMRs, including at this region,^18^ could overestimate the amount of 5mC present. Additionally, the presence of (and/or changes in) 5hmC at the same loci could complicate any interpretation of downstream effects on gene expression, since the role of 5hmC at imprinted DMRs is unclear.

The strengths of this study are the use of methods specific for the detection of 5mC and 5hmC and the inclusion of babies with birth weights across the normal range. Limitations of our study include that we were unable to quantify the relative amounts of 5mC and 5hmC at each locus; however, now that we have identified the presence of these marks at imprinted DMRs in placenta, future studies utilizing techniques such as Tet-assisted bisulfite (TAB) sequencing or oxidative bisulfite sequencing will allow more specific quantitation of 5mC/5hmC at individual CpGs. Such studies would permit genome-wide analysis, rather than the more limited approach of studying 5mC and 5hmC at specific DMRs known to have an impact on birth weight. We used placental DNA in our analysis, and studies that utilize available tissue from the offspring (cord blood, umbilical cord, buccal cells) would provide additional information as to mechanisms accounting for offspring disease risk.^45^ Our study included relatively low numbers of women who smoked and this means that although we were able to identify smoking as a confounder for IGF2 expression, we were not powered to undertake formal testing of mediation. We did not have access to placental weights for this study, which would have been useful for further analysis of any relationship between placenta weight, gene expression, and DNA methylation. Finally, further work to relate changes in DNA methylation, gene, and protein expression would also be useful in determining the underlying mechanisms of disease.

In conclusion, we show that for 2 imprinted loci, placental gene expression and 5mC/5hmC patterns associate with size at birth. We also show that 5hmC is present at some imprinted loci and suggest that studies aimed at describing DNA methylation in placenta should consider using specific techniques that distinguish between different DNA modifications and permit their accurate quantification. This would facilitate our further understand of the mechanisms that underpin fetal and placental growth, which, by extension, might be important in determining later disease risk.
